# Etoricoxib-Induced Fixed Erythema

**DOI:** 10.3390/jcm14238504

**Published:** 2025-11-30

**Authors:** Corina Porr, Dana M. Harris, Anca Vidrighin, Alina Catana, Cosmina Diaconu, Emi M. Preda, Mirela L. Popa, Elena C. Berghea

**Affiliations:** 1 Allergology Department, Faculty of Medicine, Lucian Blaga University, 550169 Sibiu, Romania; corina_sibiu@yahoo.com; 2 Internal Medicine Department, Mayo Clinic, Jacksonville, FL 32224, USA; danamcharris@yahoo.com; 3 Department of Paediatrics, Faculty of Medicine, Lucian Blaga University, 550169 Sibiu, Romania; 4 Hematology Department, Faculty of Medicine, Lucian Blaga University, 550169 Sibiu, Romania; alinabrabete@yahoo.com; 5 Department of Nursing and Dental Medicine, Faculty of Medicine, Lucian Blaga University, 550024 Sibiu, Romania; 6 Department of Radiology, “Carol Davila” University of Medicine and Pharmacy, 050474 Bucharest, Romania; emi.preda@umfcd.ro; 7 Department of Radiology and Medical Imaging, “Foișor” Clinical Hospital of Orthopaedics, Traumatology and Osteoarticular TB, 021382 Bucharest, Romania; 8 Faculty of Medicine, Lucian Blaga University, 550169 Sibiu, Romania; liviamirelapopa@yahoo.com; 9 Department of Paediatrics, Carol Davila University of Medicine and Pharmacy, 020021 Bucharest, Romania; camelia.berghea@umfcd.ro; 10 Department of Clinical Allergology and Clinical Immunology, Marie S. Curie Emergency Children’s Clinical Hospital, 077120 Bucharest, Romania

**Keywords:** fixed drug eruption, etoricoxib, cutaneous drug reaction, patch test, oral challenge test, non-steroidal anti-inflammatory drugs

## Abstract

**Background:** Fixed drug eruption (FDE) is a non-immediate, CD8+ T cell–mediated hypersensitivity reaction characterized by well-demarcated erythematous–violaceous plaques that recur at the same site after re-exposure to the causative drug. Although NSAIDs and antibiotics are the most common triggers, various other medications may induce FDE, and genetic susceptibility has been linked to specific HLA alleles. **Methods:** We conducted a clinical evaluation supported by patch testing, oral drug provocation, and assessment of therapeutic alternatives to identify the causative agent and confirm delayed-type hypersensitivity. **Results:** We report the case of a 53-year-old woman with essential hypertension, autoimmune thyroiditis, and renal lithiasis who developed well-demarcated erythematous plaques with central vesiculation and moderate pruritus on the dorsal hand and posterior calf approximately 8 h after ingestion of a 60 mg etoricoxib tablet. Patch testing was negative, while oral challenge confirmed etoricoxib-induced FDE; celecoxib was subsequently evaluated as a potential safe alternative. **Conclusions:** This case underscores the importance of an integrated diagnostic approach—including careful history, clinical examination, and confirmatory testing—to accurately diagnose delayed cutaneous drug reactions and to identify safe therapeutic options for patients.

## 1. Introduction

Adverse drug reactions include both dose-dependent reactions (which are predictable and stem from the drug’s pharmacological properties) and dose-independent reactions (which are unpredictable and influenced by patient-specific factors as well as the drug itself). The latter category includes drug eruptions, which represent roughly 25–30% of all adverse drug reactions [1]. FDEs make up about 14–22% of all cutaneous drug reactions. Lesions may recur at the same site within a few hours after re-exposure to the drug. This phenomenon is referred to as recurrence at identical sites [2]. FDE most commonly presents as a single lesion; however, multiple lesions may appear on non-adjacent areas of the body, representing generalized FDE [3].

## 2. Materials and Methods

Drugs administered orally are the most common cause of FDE [4]. Less common drug exposures may be topical or intravaginal. FDE is a non-immediate drug hypersensitivity reaction manifested by the appearance of one or more isolated, well-demarcated erythematous-violaceous plaques, which appear in the same place if the patient is re-exposed to the drug that caused the reaction. Sometimes patients may experience pain or itching, and the rash may often be mistaken for insect bites, urticaria, or erythema multiforme [5].

A variety of medications can be responsible for FDE, with antibiotics and NSAIDs being the most frequent offenders. Less commonly, sedatives, anticonvulsants, antihypertensives, and several other drugs have also been identified as causes of FDE [4,6,7,8,9].

A category of drugs very rarely implicated in this condition is benzodiazepines, such as lorazepam, lormetazepam, or chlordiazepoxide [10,11].

A rare trigger of this condition is chlordiazepoxide, which was recently implicated in the case of a patient with alcohol dependence who was admitted to a specialized clinic for treatment of this addiction. Within 24 h of administration at a dose of 60 mg/day, the patient developed hyperpigmented macules on the limbs and trunk. The medication was discontinued, and specific treatment was initiated, resulting in complete healing of the lesions within one week [12].

It is important to consider that in patients undergoing alcohol withdrawal and receiving high doses of chlordiazepoxide, this drug may induce a fixed drug eruption.

A distinct category of drugs is represented by monoclonal antibodies, which have brought numerous advantages to medicine, particularly for oncology patients, through their precise action on tumor cells. A systematic review conducted this year analyzed the potential cutaneous reactions associated with monoclonal antibody therapy [13].

Monoclonal antibodies can induce various reactions and are associated with type I, II, III, and IV hypersensitivity [14,15].

The most frequently reported dermatological adverse reactions to monoclonal antibodies are SJS, TEN, FDE, and erythema multiforme (EM) [8,16,17,18,19].

It has been observed that anti-PD1 checkpoint inhibitors, such as pembrolizumab, nivolumab, and sintilimab, are monoclonal antibodies responsible for inducing severe dermatological reactions, namely SJS/TEN and EM [8].

Monoclonal antibodies associated with the induction of FDE are TNF-alpha inhibitors. Adalimumab is the most frequently reported monoclonal antibody in this category and can trigger reactions even up to 12 days after drug initiation [15].

A possible cause of FDE may be the activation of CD8+ T cells and prolonged expression of ICAM-1 in resident keratinocytes, which can lead to the release of multiple immunological mediators [20]. Additionally, TNF-alpha blockade may play an important role in this process.

In the case of monoclonal antibody use, it is suggested that patient education regarding early symptoms is important, and patients should photograph lesions both at onset and subsequently, as this can assist in monitoring their progression and guiding treatment. This approach may also be helpful for other categories of drugs that trigger FDE.

Another category of drugs widely used in clinical practice is fluoroquinolones, antibiotics primarily used for urinary, respiratory, cutaneous, or gastrointestinal bacterial infections. A case of FDE was reported in a patient who was prescribed therapy with levofloxacin [21].

Eight hours after initiating levofloxacin therapy for a respiratory infection, the patient developed lesions characteristic of FDE on the chin and lower limbs. The medication was discontinued, leading to lesion resolution, although residual hyperpigmentation persisted. Among fluoroquinolones, cases of FDE have also been reported following ciprofloxacin use [22].

A category of antibiotics that can frequently cause FDE is chemotherapeutics, particularly trimethoprim-sulfamethoxazole, used for respiratory, gastrointestinal, and urinary infections. In addition to fixed drug eruption, it may cause other adverse effects, such as anaphylactic shock, rare and frequent skin rashes, urticaria, fever, neutropenia, thrombocytopenia, and SJS/TEN [23].

Lesions caused by trimethoprim-sulfamethoxazole are located around the mouth, on the trunk, and on the limbs [24], with most patients presenting multiple cutaneous lesions (96.7%), as well as mucosal involvement (66.7%). Additionally, 26.7% of patients exhibited genital lesions [25], with male patients presenting lesions on the penile prepuce [26]. This is attributed to the presence of the drug in the urethra. In Table 1, the main groups of medications that can induce FDE are presented.

A link has been noted between specific forms of drug-induced hypersensitivity reactions and human leukocyte antigens (HLA). In Vietnam, a strong association exists between the HLA-A02:07 allele and FDE triggered by trimethoprim-sulfamethoxazole. Individuals who possess the HLA-A02:07 allele are 3.33 times more likely to develop trimethoprim-sulfamethoxazole-induced FDE than those who do not carry this allele [27].

Other correlations have also been observed between HLA alleles and other dermatological conditions, namely toxic epidermal necrolysis (TEN) and Stevens–Johnson syndrome (SJS).

An important correlation in the Chinese population with SJS/TEN has been identified between aromatic anticonvulsants—including carbamazepine, phenytoin, oxcarbazepine, and lamotrigine—and the HLA-B15:02 allele [28]. Additionally, HLA-B58:01 has been associated with allopurinol administration in this population [29,30].

This relationship between HLA-B15:02 and carbamazepine has also been documented in Vietnamese, Thai, Malaysian, and South Indian patients with SJS/TEN, but not in individuals from Japan, Korea, or Europe [31,32]. In European patients, a link has been reported between HLA-B57:01 and abacavir-induced SJS/TEN [33], and between HLA-A*31:01 and carbamazepine-induced SJS/TEN [34].

In the Japanese population, HLA-B15:11 has been shown to predispose to carbamazepine-induced SJS/TEN, while HLA-A02:06 has been associated with acetaminophen-induced SJS/TEN [35].

A distinct category of patients comprises those with darker skin who develop fixed drug eruption (FDE) [36].

Most often, these patients are diagnosed with inflammatory or infectious dermatoses, such as chronic urticaria, sarcoidosis, bullous pemphigoid, eczema, and tinea corporis. In such cases, the diagnosis was established based on skin biopsy samples, which revealed dermatitis with eosinophils and melanophages [8].

Etoricoxib offers strong anti-inflammatory effects along with a favorable safety profile. Although its involvement in various skin reactions has been documented—similar to other drugs in its class—the frequency is considerably lower. Reported cases include urticaria and angioedema, generalized exanthematous pustulosis, exudative erythema multiforme, toxic epidermal necrolysis, and fixed drug eruption [37,38,39,40,41]. FDE is a delayed type of IVc hypersensitivity reaction in which the activation of resident CD8+ T cells causes damage to the basal layer of the skin. Mediators are released and other immune cells are recruited, ultimately leading to damage of keratinocytes and melanocytes [4,42,43,44].

FDE represents a delayed-type hypersensitivity reaction driven by CD8+ T cells. During the first eight hours following exposure to the offending agent, these CD8+ T cells become activated and release proinflammatory cytokines—TNF-alpha, interferon-γ, perforin, and granzyme B—which act on melanocytes and basal keratinocytes. The resulting melanocyte damage causes melanin to be released into the dermis, a process regulated by interleukin (IL)-10, which is produced through the activation of FoxP3+CD4+ regulatory T cells within the initial 24 h.

Once the causative drug is stopped, the basal layer of the epidermis begins to regenerate, while the inflammatory cells undergo apoptosis. During this repair process, dermal macrophages phagocytose the extravasated melanin and persist at the lesion site, resulting in residual hyperpigmentation. Basal keratinocytes release IL-15, which supports the development of resident memory CD8+ T cells that contribute to the recurrence of FDE at the same anatomical site [4,45,46]. 

Upon first administration of the drug, FDE lesions may appear within one week; however, after subsequent administrations of the same drug, the plaques develop within 8–24 h [11].

In FDE, lesions typically appear on the extremities (hands and feet) and in areas with thin skin (lips, genitalia, and the perianal region), but they can occur on any part of the body. Pruritus and pain may precede the lesions, or the condition may be asymptomatic [4].

Clinically, FDE’s characteristic is the recurrence of single or multiple cutaneous and/or mucosal lesions as well-defined, round or oval, purplish or brownish patches. Sometimes blisters may appear, and/or bullae may develop into purpuraceous discoid erythema. Residual hyperpigmentation usually occurs in the affected sites, which may last up to several months and may recur at the same or different site on re-administration of the drug. The post-inflammatory hyperpigmentation darkens with each recurrence.

FDE can be diagnosed clinically, but the histopathological examination of a skin biopsy reveals interface dermatitis with melanophages and eosinophils.

In the early stages, one can observe isolated apoptotic keratinocytes, vacuolar degeneration, dermal edema, and a superficial perivascular inflammatory infiltrate composed of lymphocytes and eosinophils. The vesicles are subepidermal. In the late lesions, melanophages are observed in the upper dermis, corresponding to post-inflammatory hyperpigmentation.

There are several conditions that should be considered for the differential diagnosis, depending on the clinical appearance and location. Among these, we mention: bullous reaction to insect bites, bullous pemphigoid and other autoimmune bullous diseases, erythema multiforme, Stevens–Johnson syndrome, and toxic epidermal necrolysis (Lyell’s syndrome). In the case of oral lesions, the differential diagnosis includes herpetic infection (herpes simplex), aphthous ulcers, or autoimmune bullous diseases of the oral mucosa.

Complications of fixed drug eruptions may include the appearance of vesicles and erosions on the lesions, sometimes accompanied by local discomfort or pain. In some cases, cross-reactions with other drugs from the same pharmacological class may occur, increasing the risk of lesion recurrence. The generalized bullous form of fixed drug eruption can be complicated by fluid loss, electrolyte imbalances, and secondary infections, situations that require prompt medical treatment and careful monitoring [47].

**Figure 1:**
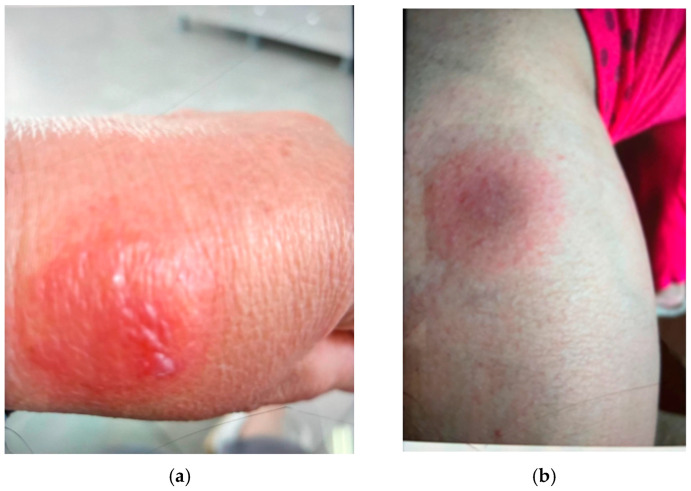
Representative images of FDE in a 53-year-old female patient treated with etoricoxib. A rash consisting of round-oval, well-demarcated, intensely erythematous plaques with central vesiculosis, localized on the dorsal aspect of the left hand (**a**) and back of the calf (**b**), 8 h post exposure to etoricoxib.

We present the case of a 53-year-old female patient, known to have essential hypertension, autoimmune thyroiditis, hypothyroidism for which she is undergoing chronic therapy (Calcium channel blocker—Amlodipina and Levothyroxine), renal lithiasis, she presented for the appearance of a rash consisting of round-oval, well-demarcated, intensely erythematous plaques, some with central vesiculosis, associated with moderate intensity cutaneous pruritus, localized on the dorsal aspect of the left hand (Figure 1a), back of the calf (Figure 1b).

The rash started about 8 h after taking an etoricoxib tablet 60 mg. The patient reported that she developed two similar lesions, which spontaneously resolved, a year earlier, also after taking the same drug. A general physical examination of the patient revealed the following: relatively good general condition, presence of adipose connective tissue in excess, intermittent pain with inflammatory character at the lumbosacral spine, normal chest, blood pressure of 170/120 mmHg (the patient did not take her prescribed antihypertensive therapy), HR (heart rate) = 68 bpm, rhythmic heart sounds, synchronous with the pulse wave. A direct clinical examination established the presence on the limbs of multiple round-oval, intensely erythematous, well-demarcated plaques, some with central vesicular aspect, with diameters ranging from 1 cm to 8 cm. The patient was prescribed a therapy with topical cortisone preparations for two days (Hydrocortisone cream 1%), followed by application of local cortisone therapy, twice a day, and antihistamines (desloratadine 5 mg once a day). This new treatment was associated with the pre-existing patient’s chronic therapy. Since the FDE evolution was favorable, the patient was advised to no longer take etoricoxib.

**Figure 2:**
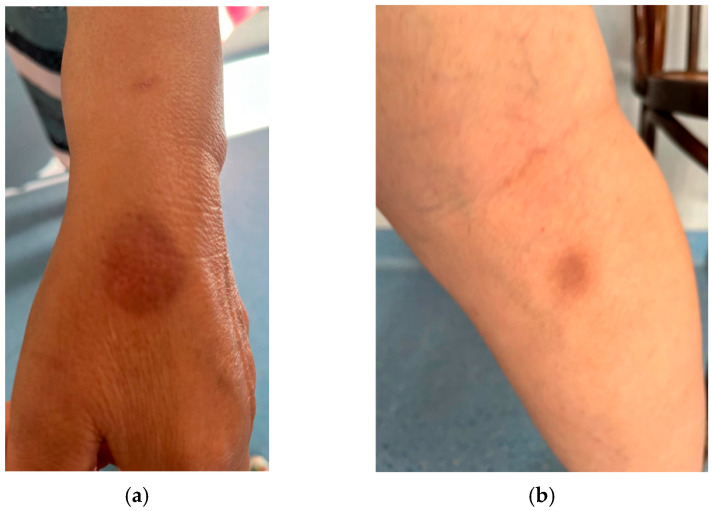
Images of FDE on hand and leg in a 53-year-old female patient treated with etoricoxib. Round oval hyperpigmentation plaques, well-delimited on the hand (**a**) and back of leg (**b**).

After two months, the patient still presented well-delimited, round, oval hyperpigmentation plaques of 3 cm diameter on the hand (Figure 2a) and leg (Figure 2b). We would like to clarify that in our clinic, each patient is informed about the risks of every procedure, including the use of images for publication, and these images are only published after we obtain the patient’s consent.

Since the patch test is recommended as a first diagnostic step for FDE, we decided to perform the test with etoricoxib (30% (*w*/*w*) in petrolatum), both on healthy skin and on an area that remained hyperpigmented. The patch test was prepared as a 30% (*w*/*w*) suspension of the drug in petrolatum, in accordance with available literature on non-immediate drug eruptions. The skin test was read at 48 and 72 h, respectively, and it was negative in both areas. Since the patient did not have a generalized post-drug fixed erythema, we considered that the risk of severe adverse reactions is not high and decided to perform the open oral challenge test. It was performed in a hospital, under continuous monitoring, with resuscitation equipment immediately available, and the patient provided written informed consent after being informed of all risks. The oral provocation test remains the most reliable method for establishing the diagnosis. There are no guidelines for drug testing in these cases, and if patients do not have many cutaneous lesions and the standard-dose provocation test is negative, meaning that no cutaneous reactions occur, the drug dose can be doubled. In generalized forms, the provocation test is contraindicated [48].

**Figure 3:**
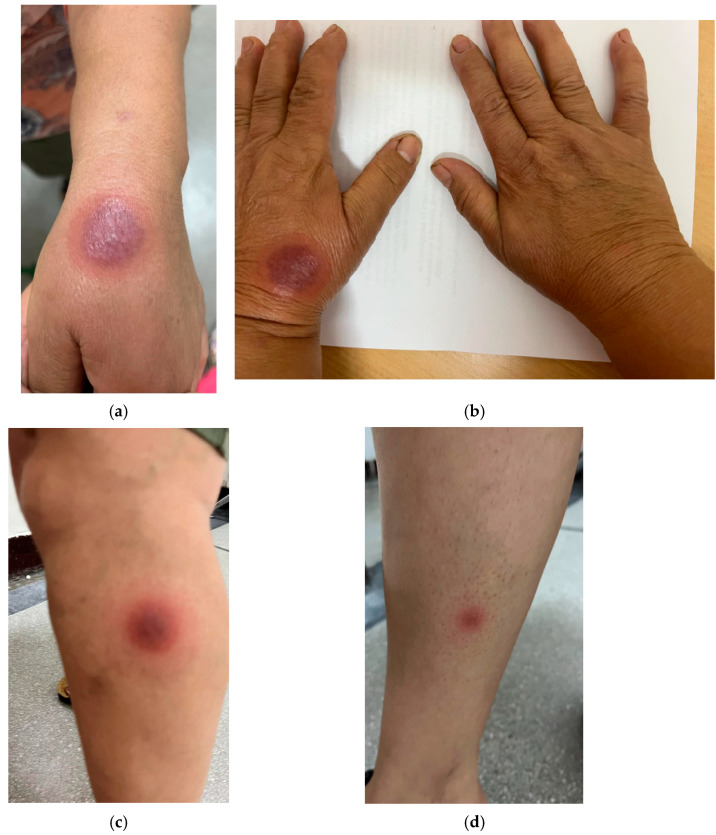
Images of FDE in a 53-year-old female patient treated with etoricoxib. (**a**,**b**) Well-demarcated, erythematous, round oval plaques on the left hand; (**c**) one erythematous plaque with a few vesicles situated on the upper third of the left calf; (**d**) one plaque on the forearm.

For the open oral challenge test, we started with 10% of the prescribed 60 mg dose (i.e., 6 mg of etoricoxib), and since no local or systemic changes occurred, we continued administering 10% of the daily dose (6 mg etoricoxib) every 30 min. Then, we administered 20% of the 60 mg etoricoxib dose (12 mg etoricoxib) every 30 min four more times, monitoring the patient throughout this period. Eight hours after the administration of the therapeutic dose, the patient developed again, on the same sites (Figure 3a,b), well-demarcated, erythematous, round oval plaques and two additional plaques: one erythematous plaque with a few vesicles was situated on the upper third of the calf (Figure 3c) and the other on the forearm, with two cm diameter (Figure 3d). Again, antihistaminic and local cortisone therapy was administered as before, twice a day, with a slow, favorable evolution and persistent hyperpigmentation on the affected sites after six weeks. We would like to mention that HLA testing was not performed, as it is not available free of charge and would involve costs for the patient, and in our case, the patient refused to undergo it.

Some cases of cross-reactivity between structurally unrelated NSAIDs and between etoricoxib and celecoxib have been reported in the literature [49,50]. With the goal of offering the patient a safer alternative anti-inflammatory therapy, we set forth to conduct testing of celecoxib. Patch skin testing was performed with celecoxib on both the clear and hyperpigmented areas. We applied 30% (*w*/*w*) celecoxib in Vaseline using Finn chambers with occlusion periods of 24 h. No positive reaction to celecoxib was observed in lesional or normal skin. Since the skin patch test with etoricoxib was negative, while the open oral challenge test was positive, we decided to also perform the open oral challenge test with celecoxib. The celecoxib 100 mg oral provocation test was also performed in a hospital setting with an intravenous line and continuous monitoring. The drug was administered in 25 mg increments every 30 min, with the provocation test lasting two hours, while monitoring continued for 48 h. The patient did not exhibit any clinical manifestations, and at a seven-day follow-up, no cutaneous reactions were observed.

## 3. Discussion

For drug-induced dermatologic conditions, the diagnosis is generally made based on the medical history and physical examination. It has been previously reported that a possible genetic predisposition for FDE exists function of the HLA-B22 gene expression level [51]. Hence, an HLA-B22-based susceptibility test can also be considered as part of the diagnostic procedure. In our study, however, we were unable to test for an association between HLA-B22 and patients with FDE.

We present the case of a patient with etoricoxib-induced fixed erythema. The diagnosis was based on her medical history and a physical examination. Although the etoricoxib patch skin test was negative, a rare occurrence, we were able to confirm etoricoxib as the causative drug based on the oral challenge test, which is not routinely practiced. Etoricoxib, a selective COX-2 inhibitor, has rarely been reported as a causative agent of FDE, a cyclo-oxygenase 2 selective inhibitor (COX-2 inhibitor), rarely described as the culprit drug in FDE [52], which makes this case peculiar.

As there is a possibility of cross-reactivity between drugs of the same class, using both patch skin testing and an oral challenge test may help in this case. In this case, patch test and oral provocation test were performed on an alternative anti-inflammatory drug, celecoxib, which, fortunately, was well tolerated by the patient. On the other hand, there have not been reports of demonstrated cross-reactivity between Nimesulide and Etoricoxib, members of the non-antibiotic sulfa group, which belong to different classes of NSAIDs. However, a theoretical possibility of cross-reactivity between these two drugs has been speculated [53]. Therefore, we suggested to the patient to also test Nimesulide, but the patient declined to continue skin testing with this substitute drug.

Because the consumption of anti-inflammatory drugs is increased among elderly patients who often have chronic treatments with multiple drugs, diagnosis of FDE can be difficult. The pharmaceutical industry can also contribute to this by developing and marketing new anti-inflammatory drugs.

## Figures and Tables

**Table 1:** Drugs producing fixed eruptions.

	Examples of Agents Causing Fixed Eruption
Drugs	Antibiotics NSAIDs Sedatives Anticonvulsants Antihypertensives Benzodiazepines Monoclonal antibodies Fluoroquinolones Chemotherapeutics

## Data Availability

The original contributions presented in the study are included in the article; further inquiries can be directed to the corresponding authors.
